# Analysis and identification of the necroptosis landscape on therapy and prognosis in bladder cancer

**DOI:** 10.3389/fgene.2022.919829

**Published:** 2022-09-29

**Authors:** Zihan Zhao, Ning Jiang, Yulin Zhang, Yuhao Bai, Tianyao Liu, Tianhang Li, Hongqian Guo, Rong Yang

**Affiliations:** ^1^ Department of Urology, Nanjing Drum Tower Hospital, The Affiliated Hospital of Nanjing University Medical School, Institute of Urology, Nanjing University, Nanjing, China; ^2^ Department of Urology, Nanjing Drum Tower Hospital Clinical College of Jiangsu University, Nanjing, China

**Keywords:** bladder cancer, necroptosis, tumor immune microenvironment, prognostic signature, bioinformatics analysis

## Abstract

Bladder cancer (BLCA) is one of the most common malignant tumors of the urinary system, but the current therapeutic strategy based on chemotherapy and immune checkpoint inhibitor (ICI) therapy cannot meet the treatment needs, mainly owing to the endogenous or acquired apoptotic resistance of cancer cells. Targeting necroptosis provides a novel strategy for chemotherapy and targeted drugs and improves the efficacy of ICIs because of strong immunogenicity of necroptosis. Therefore, we systemically analyzed the necroptosis landscape on therapy and prognosis in BLCA. We first divided BLCA patients from The Cancer Genome Atlas (TCGA) database into two necroptosis-related clusters (C1 and C2). Necroptosis C2 showed a significantly better prognosis than C1, and the differential genes of C2 and C1 were mainly related to the immune response according to GO and KEGG analyses. Next, we constructed a novel necroptosis-related gene (NRG) signature consisting of *SIRT6*, *FASN*, *GNLY*, *FNDC4*, *SRC*, *ANXA1*, *AIM2*, and *IKBKB* to predict the survival of TCGA-BLCA cohort, and the accuracy of the NRG score was also verified by external datasets. In addition, a nomogram combining NRG score and several clinicopathological features was established to more accurately and conveniently predict the BLCA patient’s survival. We also found that the NRG score was significantly related to the infiltration levels of CD8 T cells, NK cells, and iDC cells, the gene expression of CTLA4, PD-1, TIGIT, and LAG3 of TME, and the sensitivity to chemotherapy and targeted agents in BLCA patients. In conclusion, the NRG score has an excellent performance in evaluating the prognosis, clinicopathologic features, tumor microenvironment (TME), and therapeutic sensitivity of BLCA patients, which could be utilized as a guide for chemotherapy, ICI therapy, and combination therapy.

## Introduction

Bladder cancer (BLCA) is one of the most common malignant tumors of the urinary system, and its mortality and morbidity have been increasing in recent years (Sung et al., 2020). Although the majority of new cases are non-muscular invasive tumors, once the muscular infiltration stage is entered, the 5-year survival rate drops to 36–48% (Lenis et al., 2020). At present, the first-line treatment for advanced bladder cancer is chemotherapy based on cisplatin, but about 40% of patients are still unable to benefit from it or even suffer continuous progression due to the high heterogeneity of BLCA (Witjes et al., 2021). Because of the strong immunogenicity in the tumor microenvironment (TME) of BLCA, the immune checkpoint inhibitors (ICIs) have also achieved certain therapeutic effects in the treatment of BLCA (Roviello et al., 2021). But it still faces the problem of a less than 30% response rate (de Jong et al., 2021). It is necessary to optimize the currently used therapy strategy, including chemotherapy and ICIs, before the next generation of effective antitumor treatment regimens is mature, such as adoptive cell therapy.

Whether it is cisplatin-based chemotherapy or ICIs represented by anti-PD-1/PD-L1, the main way to effectively kill tumor cells is to induce tumor cell apoptosis. Previous studies have shown that the resistance to cisplatin during treatment may be due to the resistance of tumor cells to apoptosis (Johnstone et al., 2002). Therefore, it is a feasible strategy to improve the therapeutic effect of chemotherapy and immunotherapy in BLCA by developing methods to induce non-apoptotic forms of programmed cell death as a combination therapy. Necroptosis is a novel apoptosis-independent programmed cell death with strong immunogenicity that plays a critical role in cancer patient prognosis, cancer progression and metastasis, cancer immune surveillance, and cancer subtypes (Gong et al., 2019). The lower expression of the key molecules in the necroptosis signaling pathway was associated with a poor prognosis and promoted tumorigenesis and metastasis in multiple cancers (Park et al., 2009; Ke et al., 2013; Wang et al., 2019; Li et al., 2022; Xin et al., 2022). In addition, in clinical trials of immune checkpoint inhibitor therapy, the key molecules of necroptosis, including RIPK1, RIPK3, and MLKL, were associated with the function of T cells, and the overexpression of these genes was related to better survival (Tang et al., 2020). In addition, considering the tolerance of tumor cells to apoptosis is responsible for the resistance to chemotherapy, induction of necroptosis acts as an effective strategy to address the issue of apoptosis resistance during chemotherapy, and a variety of anticancer drugs have been developed to induce necroptosis (Fulda, 2014). Given the critical role of necroptosis in cancer development and treatment, necroptosis has emerged with a great potential for cancer therapy by improving the therapeutic effect of chemotherapy and immunotherapy in BLCA.

However, there are few studies focusing on necroptosis of BLCA as far. In this study, we analyzed the expression of necroptosis-related gene (NRG) in 335 BLCA patients from The Cancer Genome Atlas (TCGA) database. The survival analysis, TME analysis, and treatment efficacy analysis of NRGs were used. Moreover, a prognostic signature (NRG score) and nomogram model based on the NRG score were constructed. The validation with independent cohorts showed that the NRG score provided accurate prognostic prediction for BLCA patients. In conclusion, we have verified that the NRG score is significantly correlated with TME and prognosis of BLCA patients, which suggests that targeting necroptosis through various drugs that induce or manipulate necrotic pathways in BLCA may be considered an adjuvant therapy strategy.

## Materials and methods

### Access to datasets and patients information

Transcription RNA sequencing and clinical information of patients in the BLCA cohort were publicly obtained from TCGA database (https://portal.gdc.cancer.gov/). Transcription RNA sequencing included 414 BLCA tissues and 19 normal bladder tissues. The RNA-sequencing data in this cohort was downloaded as fragments per kilobase of transcript per million mapped reads (FPKM). When an individual gene symbol contained more than one Ensembl ID in RNA sequencing data, gene expression was annotated averagely. We removed 98 samples with incomplete clinical information, and finally 335 patients were included in the subsequent study. We used an independent cohort (GSE13507 and GSE31684) containing merged 258 primary bladder cancer samples for external validation. The corresponding RNA-seq data and the survival information of patients were obtained from the Gene Expression Omnibus (GEO, http://www.ncbi.nlm.nih.gov/geo).

### Retrieval of necroptosis-related genes

We obtained 598 necroptosis-related genes from the GeneCards (https://www.genecards.org/). Using univariate Cox analysis, 17 genes associated with bladder cancer prognosis (*p* < 0.01) were identified and used as NRGs for the subsequent analysis.

### Consensus clustering for NRGs and functional enrichment analysis

Based on the gene expression of 17 NRGs in TCGA cohort, unsupervised clustering analysis was applied to determine the number of clusters. Finally, consensus clustering analysis in TCGA cohort identified two clusters using the “ConsensusClusterPlus” package. The “ggplot2” and “limma” packages were used to construct a principal component analysis (PCA). We analyzed clinicopathological differences between the two clusters with the chi-squared test. The “survival” package was applied to compare survival differences between two clusters. Gene Ontology (GO) and Kyoto Encyclopedia of Genes and Genomes (KEGG) analyses were performed by the “clusterProfiler,” “org.Hs.eg.db,” “enrichplot,”, and “GOplot” packages to conduct functional annotation of the genes with different expression (false discovery rate (FDR) < 0.05 and log2 fold change (FC) > 1) in two clusters. The analysis results were visualized through the “ggplot2” package.

### Construction and validation of the NRG prognostic signature

Based on 17 bladder prognosis-related NRGs that have been obtained by univariate Cox analysis, we performed the least absolute shrinkage and selection operator (LASSO) using the “glmnet” package. Subsequently, we used the “survival” and “MASS” packages in R to choose a model using multivariate Cox analysis which was operated in a stepwise algorithm filtered by the AIC value to identify the NRG risk signature. The NRG risk score formula was obtained as follows:

Risk score = Σ (exp Genei × coefficient Genei).

Patients in TCGA cohort were classified into high-risk and low-risk groups based on the median score. We used the “survival” and “survminer” packages to perform Kaplan–Meier analysis. The “timeROC” package in R was used for the analysis of the receiver operating characteristic (ROC) curve and to calculate the area under the ROC curves (AUC). We used GSE13507 and GSE31684 datasets as the validation cohort to validate the prognostic value of the NRG risk signature. Patients’ risk scores were calculated using the same formula as mentioned previously, and the same cutoff criteria were used to classify the patients into low-risk and high-risk groups. Then, Kaplan–Meier survival analysis and ROC curve analysis were performed on the validation cohort to assess the prognostic value. Clinicopathological data differences between the two risk groups were assessed using the chi-squared test and presented by a heatmap. In addition, univariate and multivariate Cox proportional hazards regression analyses were performed to evaluate whether the risk score and clinicopathological variables were independent prognostic factors for overall survival.

### Establishment of the nomogram model

We used NRG risk scores and clinicopathological factors to build a nomogram model to provide more accurate prognosis prediction for BLCA patients. The “rms” package in R helped to establish a nomogram for predicting prognosis. Also, we established calibration curves to test whether predicted survival was consistent with actual survival using the “rms” package.

### Gene set enrichment analysis between NRG risk groups

Gene set enrichment analysis (GSEA, http://www.broad.mit.edu/gsea/) is a computational method that determines whether an a priori defined set of genes shows statistically significant, concordant differences between two biological states. We performed GO-GSEA and KEGG-GSEA analyses between high-risk and low-risk groups using GSEA software (version 4.1.0). We chose gene set databases as “pub/gsea/gene_sets/c5. go.v7.5.1. symbols.gmt” and “pub/gsea/gene_sets/c2. cp.kegg.v7.5.1. symbols.gmt”.

### Immune checkpoint and immune enrichment analysis

We explored the differential expression of immune checkpoints between high-risk and low-risk groups, which may be related to the treatment responses of immune checkpoint inhibitors. In order to assess the tumor immune microenvironment (TIME) status of BLCA, we used single sample gene set enrichment analysis (ssGSEA) in R using “GSVA,” “limma,” and “GSEABase” packages to evaluate a total of 16 congenital and adaptive immune cells as well as 13 immune-related functions.

### Evaluation of the efficacy of treatment response

We explored the value of the predictive signature in predicting the response to BLCA treatment. The “pRRophetic” package was used to perform the ridge regression algorithm to calculate the half-maximal inhibitory concentration (IC_50_). We also assessed the differences in the response to immunotherapy between high-risk and low-risk groups. We uploaded the BLCA patients’ gene expression data to the tumor immune dysfunction and exclusion website (TIDE, http://tide.dfci.harvard.edu/) and obtained the TIDE score of each patient. We also obtained immunophenoscore of TCGA-BLCA patients from the TICA website (https://www.tcia.at/home).

### Statistical Analysis

R software (version 4.1.3) and GSEA software (version 4.1.0) were used for all statistical analysis and diagram drawing. Univariate Cox analysis was used to identify NRGs associated with overall survival (OS). LASSO regression analysis and multivariate Cox analysis filtered by AIC values were used to construct a predictive signature. The survival of patients in the high-risk and low-risk groups was analyzed using the Kaplan–Meier method and log-rank test. The Wilcoxon test was used to identify the significance of the difference between the two risk groups. A *p*-value < 0.05 was set as a statistically significant standard.

## Results

### Consensus clustering of necroptosis-related patterns in BLCA

**FIGURE 1:**
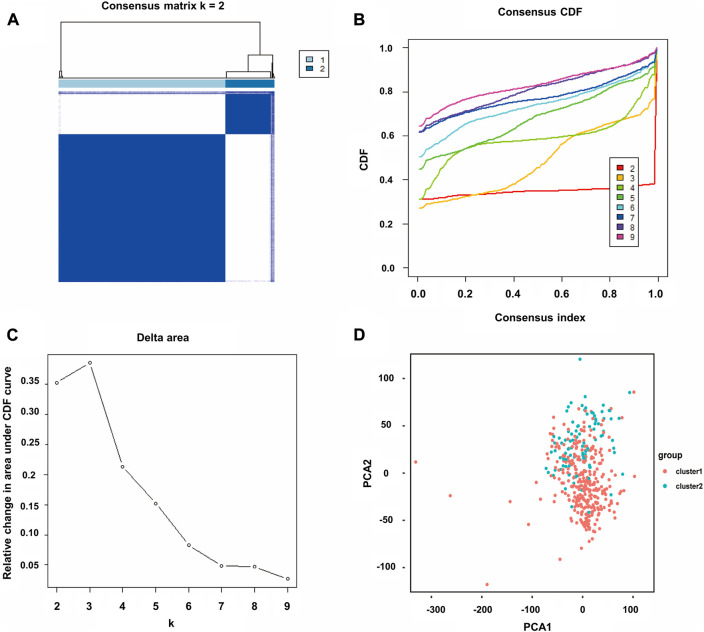
Consensus clustering of BLCA. **(A)** Correlation between subgroups when consensus matrix number k = 2. **(B)** Cumulative distribution function (CDF) is displayed for k = 2–9. **(C)** Relative change in the area under the CDF curve for k = 2–9. **(D)** Principal component analysis (PCA) of the RNA-seq data. Red dots represent cluster 1, and cyan dots represent cluster 2.

**FIGURE 2:**
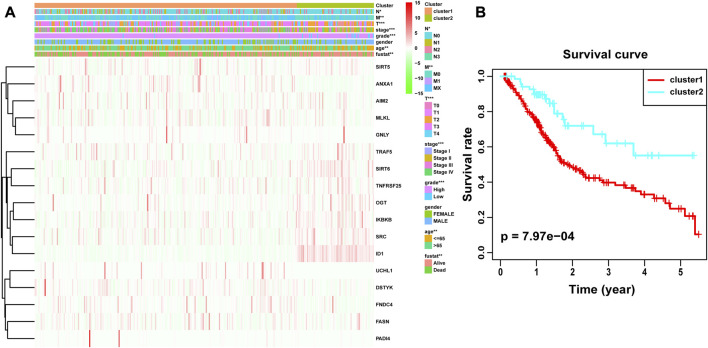
Difference in clinicopathological characteristics and overall survival between cluster 1 and cluster 2. **(A)** Heatmap and clinicopathological features of these two clusters. **(B)** Kaplan–Meier survival analysis between cluster 1 and cluster 2. *p < 0.05; **p < 0.01; ***p < 0.001.

**FIGURE 3:**
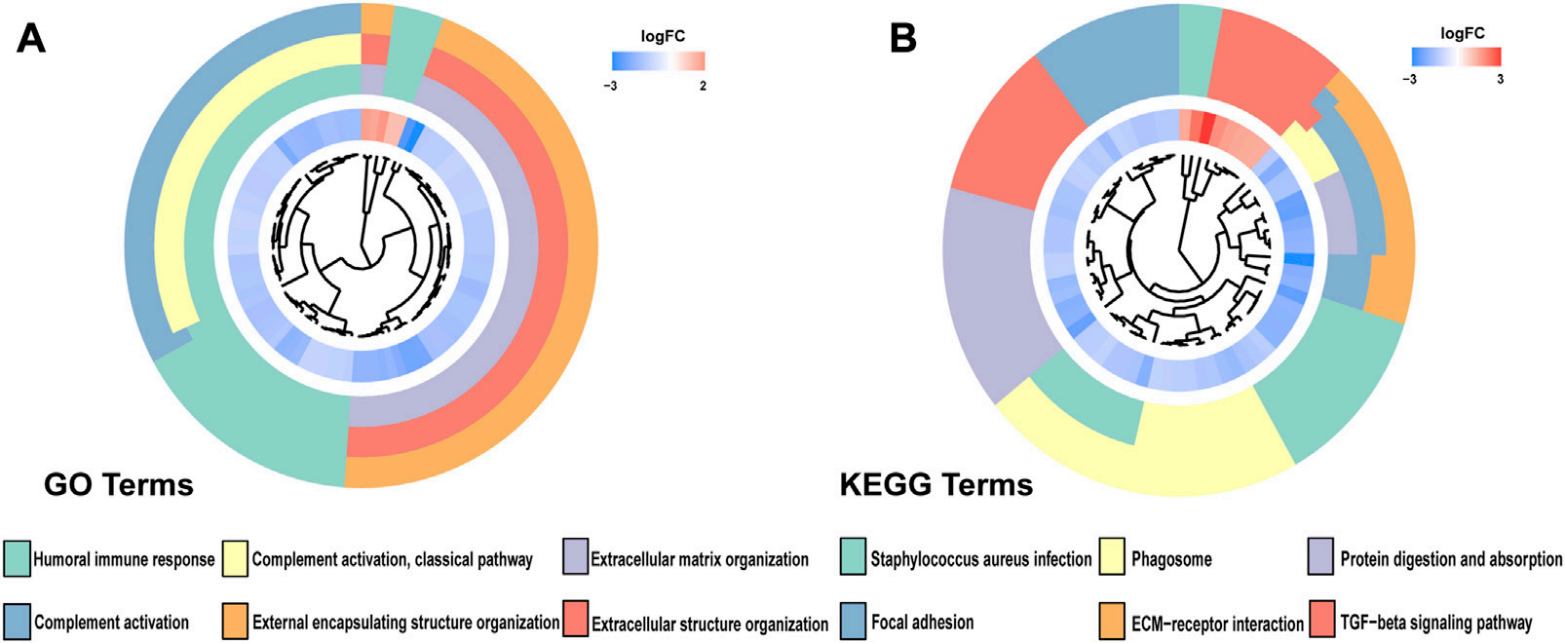
**(A)** Gene Ontology (GO) and **(B)** Kyoto Encyclopedia of Genes and Genomes (KEEG) analyses of DEGs between two clusters.

Through univariate Cox analysis of gene expression and survival data of BLCA patients in TCGA cohort, we obtained 17 bladder cancer prognosis-related NRGs (Table 1). To further explore the correlation between necroptosis-related patterns and survival and clinicopathological data of BLCA patients, we divided BLCA patients into subgroups based on their gene expression. Based on the results of consensus clustering, patients in TCGA cohort could be divided into two distinct and non-overlapping clusters (Figures 1A–C). We performed PCA analysis (Figure 1D) on the two clusters and found that cluster 1 is mainly distributed in the lower position and cluster 2 is mainly distributed in the upper position of the PCA diagram. The PCA plot showed a significant distinction between cluster 1 and cluster 2. As shown in the figure, there were significant differences in age (*p* < 0.01), pathological grade (*p* < 0.001), pathological stage (*p* < 0.001), T-stage (*p* < 0.001), M-stage (*p* < 0.01), and N-stage (*p* < 0.05) between the two groups (Figure 2A). In addition, advanced clinicopathological parameters were mainly concentrated in cluster 1. When referring to the difference in survival between the two groups, the Kaplan–Meier analysis (Figure 2B) showed that patients within cluster 1 had worse survival than those within cluster 2 (*p* = 0.003). According to the results of the GO analysis, upregulated genes were mainly enriched in humoral immune response, complement activation-classical pathway, extracellular matrix organization, extracellular structure organization, complement activation, and external encapsulating structure organization (Figure 3A). The results of the KEGG analysis showed that these upregulated genes were significantly enriched in *Staphylococcus aureus* infection, phagosome, protein digestion and absorption, TGF-beta signaling pathway, focal adhesion, and ECM–receptor interaction (Figure 3B).

**TABLE 1:** Seventeen necroptosis-related genes associated with bladder cancer prognosis identified by univariate analysis.

Gene	HR	HR.95L	HR.95H	*p*-value
*MLKL*	0.660267	0.495817	0.879261	0.004505
*UCHL1*	1.129145	1.030412	1.237338	0.009277
*SIRT6*	0.474079	0.319307	0.703870	0.000214
*FASN*	1.347467	1.123951	1.615432	0.001270
*GNLY*	0.797385	0.692176	0.918584	0.001711
*TRAF5*	0.640967	0.462229	0.888820	0.007663
*FNDC4*	1.271419	1.093672	1.478055	0.001776
*SIRT5*	0.515172	0.334930	0.792410	0.002535
*SRC*	0.766829	0.630499	0.932636	0.007855
*ANXA1*	1.218066	1.095015	1.354946	0.000283
*OGT*	0.693606	0.540500	0.890081	0.004040
*ID1*	0.859464	0.781176	0.945599	0.001885
*PADI4*	1.699223	1.185152	2.436279	0.003926
*AIM2*	0.857089	0.779962	0.941842	0.001349
*TNFRSF25*	0.678689	0.532755	0.864596	0.001702
*DSTYK*	1.778230	1.198776	2.637778	0.004221
*IKBKB*	0.556251	0.401806	0.770060	0.000409

### Establishment of the NRG score in TCGA-BLCA cohort

**FIGURE 4:**
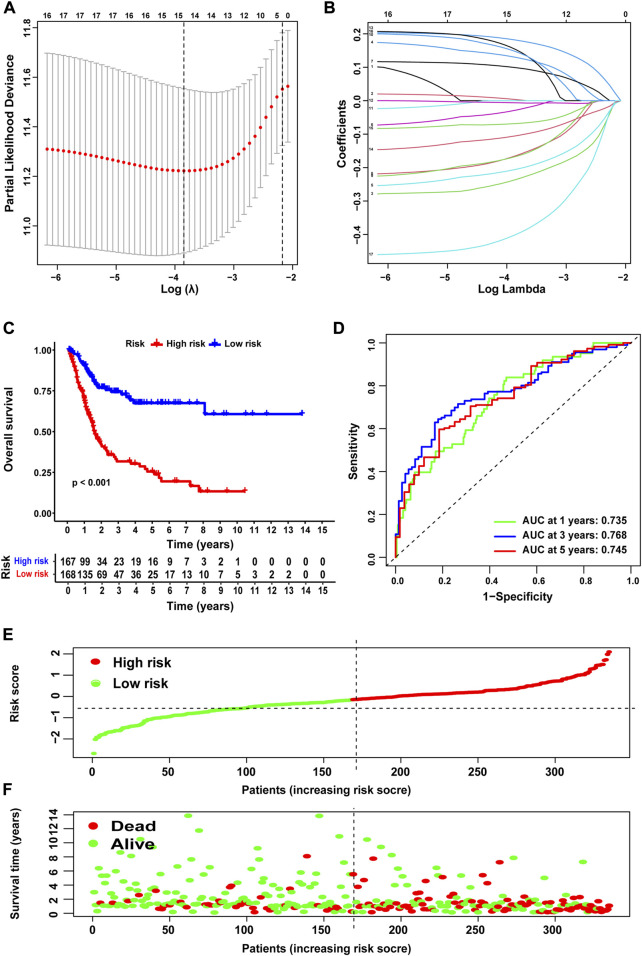
Construction of NRG score and prognosis analysis based on TCGA cohort. **(A,B)** LASSO Cox regression analysis. **(C)** Kaplan–Meier survival analysis between NRG score-defined groups. **(D)** Time-dependent ROC curves and AUCs at 1-, 3-, and 5-year survival for the 0 NRG score. **(E)** NRG score distribution. **(F)** Survival status map.

**FIGURE 5:**
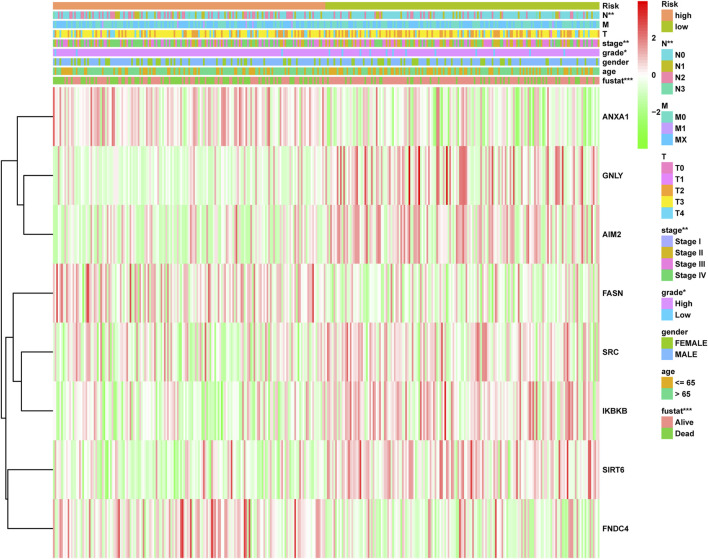
Distribution heatmap of NRG score and clinicopathological variables in the high- and low-risk groups. *p < 0.05, **p < 0.01, ***p < 0.001.

To accurately and effectively predict the clinical survival rate of BLCA patients, we established an NRG prognostic signature from TCGA database by LASSO Cox regression analysis and multiple stepwise Cox regression analysis filtered by AIC values (Figures 4A,B). Finally, we identified the optimal prognostic signature containing 8 NRGs, namely, *SIRT6*, *FASN*, *GNLY*, *FNDC4*, *SRC*, *ANXA1*, *AIM2*, and *IKBKB*. We also obtained a quantitative indicator: NRG score = (−0.1539 × *SIRT6* expression) + (0.2289 × *FASN* expression) + (−0.1683 × *GNLY* expression) + (0.1683 × *FNDC4* expression) + (−0.1851 × *SRC* expression) + (0.2437 × *ANXA1* expression) + (−0.0918 × *AIM2* expression) + (−0.3567 × *IKBKB* expression). According to the aforementioned calculation formula, we divided BLCA patients into low-risk group (N = 168) and high-risk group (N = 167) based on the median NRG score to evaluate the prognostic value of the NRG score. The Kaplan–Meier analysis showed a worse OS in the high-risk group patients relative to the low-risk group patients (Figure 4C, *p* < 0.001). Then, we performed the ROC curves to assess the efficacy of the NRG prognostic signature for survival of BLCA patients. As shown in the picture, the area under ROC curves (AUCs) for the 1-, 3-, and 5-year OS were 0.735, 0.768, and 0.745, respectively (Figure 4D). Figure 4E shows the risk score distribution of patients with BLCA. A dot pot was used to display the survival status of each patient in this cohort (Figure 4F). We also evaluated the correlation between NRG score and clinicopathological characteristics and showed it through a heatmap (Figure 5). The high-risk group had advanced pathological grade (*p* < 0.05), pathological stage (*p* < 0.01), and N-stage (*p* < 0.01) than the low-risk group.

### Validation of the prognostic signature using the GEO database

**FIGURE 6:**
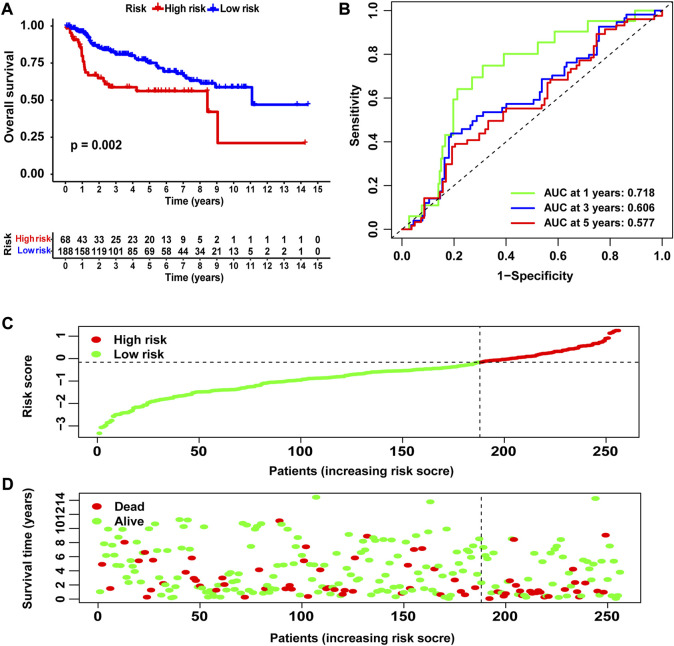
Validation of the NRG score based on the test cohort. **(A)** Kaplan–Meier survival analysis between NRG score-defined groups. **(B)** Time-dependent ROC curves of the NRG score. **(C)** NRG score distribution. **(D)** Survival status map.

We used merged GSE13507 and GSE31684 as the external validation cohort to estimate the stability and accuracy of the NRG score. After excluding two patients who survived less than 30 days, the validation cohort included 256 primary bladder cancer patients. BLCA patients were divided into low-risk group (N = 188) and high-risk group (N = 68) according to the cutoff value of TCGA cohort. We explored the prognostic value of the NRG signature in the validation cohort and obtained the results that were consistent with TCGA cohort. Patients in the high-risk group had significantly lower OS than those in the low-risk group (Figure 6A, *p* = 0.002). The AUCs for the 1-, 3-, and 5-year OS survival rates were 0.718, 0.606, and 0.577, respectively (Figure 6B). The risk score distribution and survival status plots showed similar results to those of TCGA cohort (Figures 6C and D).

### Relationship between the predictive signature and the prognosis of BLCA patients in different clinicopathological characteristics

**FIGURE 7:**
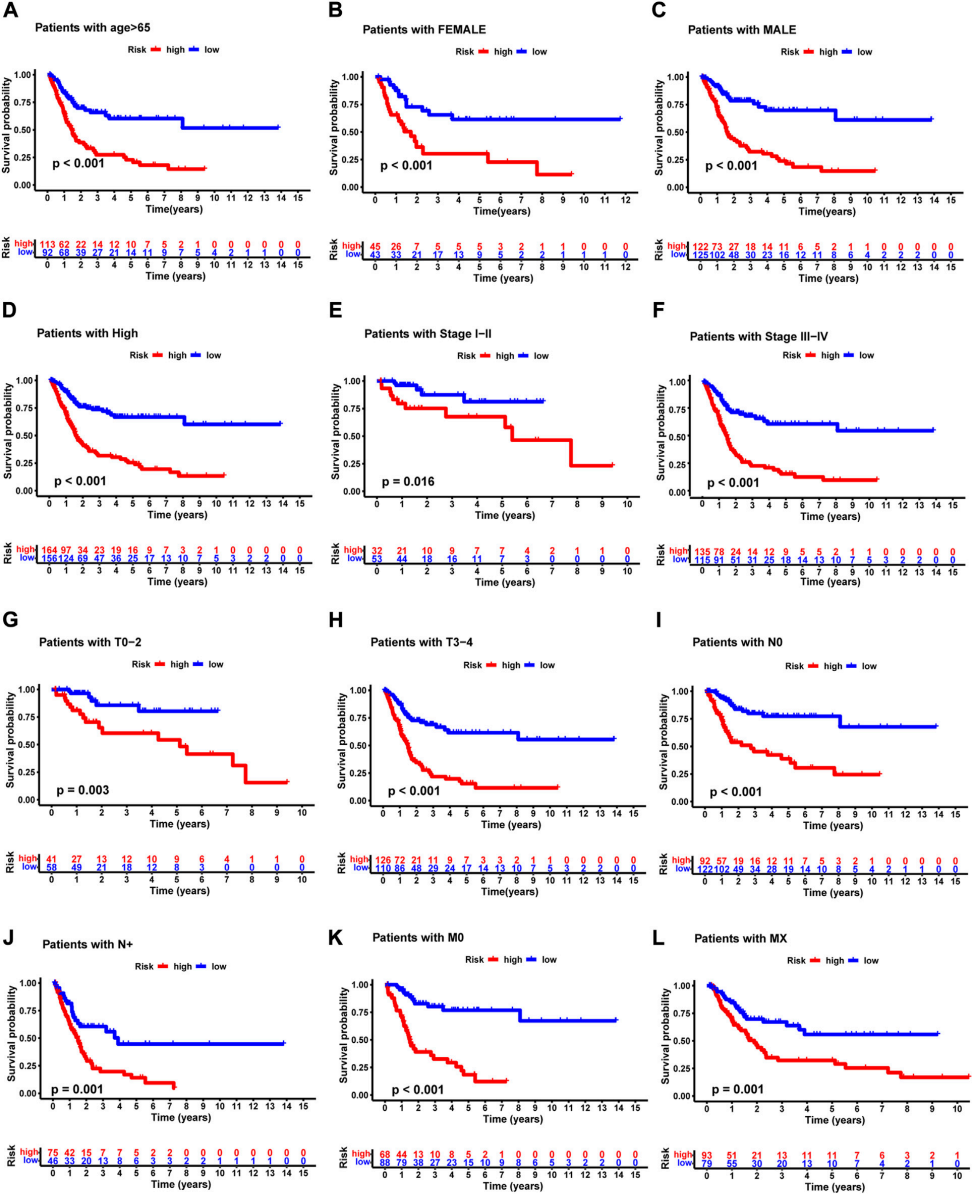
Kaplan –Meier survival curves of high- and low-risk groups among patients sorted according to different clinicopathological features. **(A)** Age. **(B,C)** Gender. **(D)** Pathological grade. **(E,F)** Pathological stage. **(G,H)** T-stage. **(I,J)** N-stage. **(K,L)** M-stage. T, tumor; N, lymph node.

We grouped the BLCA patients according to different clinicopathological features to explore the relationship between NRG score and each clinicopathological characteristic. Patients were sorted into groups according to age, gender, pathological grade, pathological stage, T-stage, M-stage, and N-stage. As shown in Figure 7, the OS of BLCA patients in the high-risk group was significantly lower than that in the low-risk group. This suggested that the NRG prognostic signature can accurately predict the prognosis of BLCA patients in different clinicopathological characteristics.

### Construction of a nomogram model based on the NRG score

**FIGURE 8:**
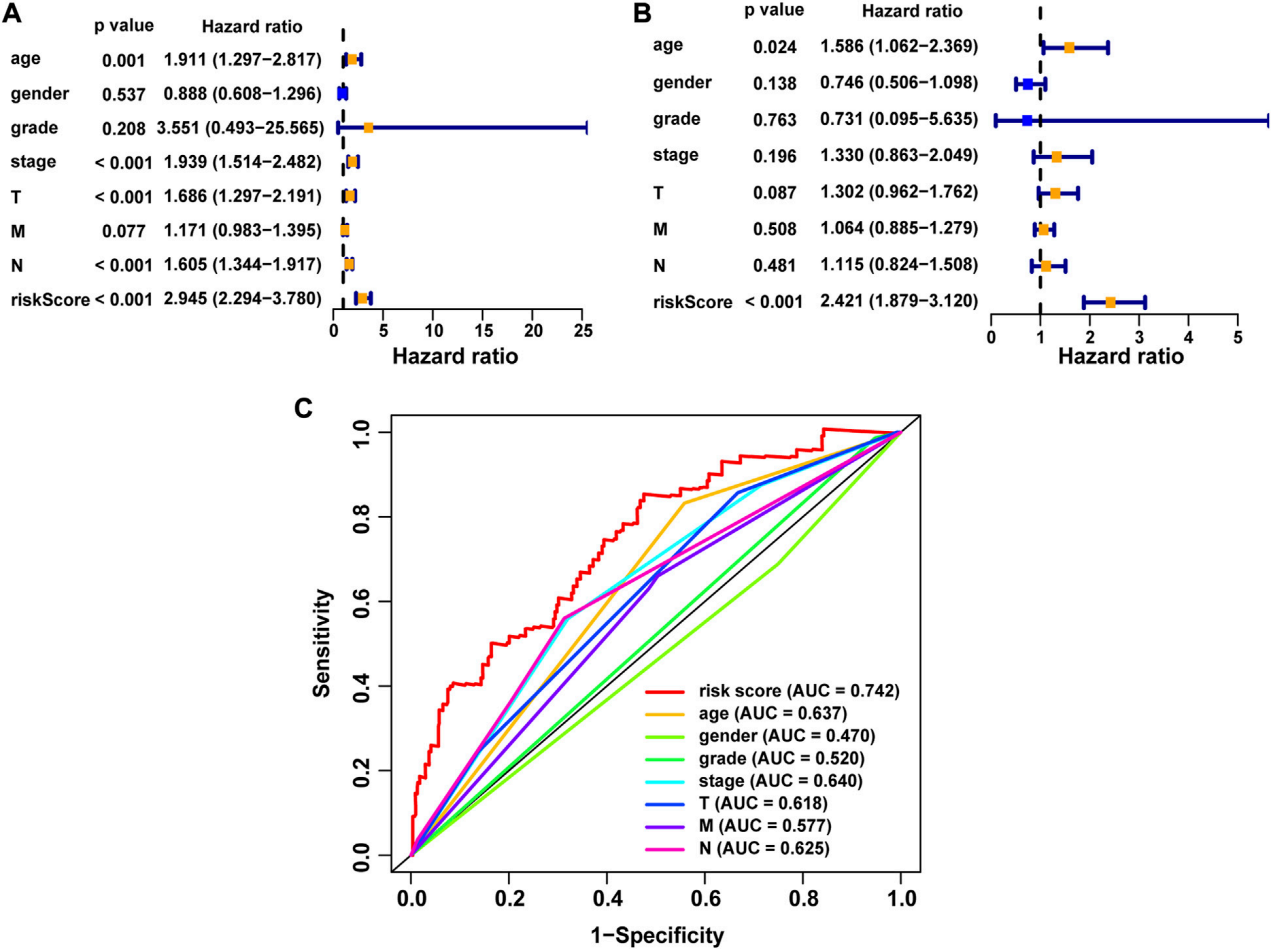
Correlation between NRG score and the prognosis of BLCA patients. **(A)** Forest plot for univariate Cox regression analysis. **(B)** Forest plot for multivariate Cox regression analysis. **(C)** ROC curve and AUCs of the risk score and clinicopathological variables. ROC, receiver operating characteristic; AUC, area under the curve; T, tumor; N, lymph node.

**FIGURE 9:**
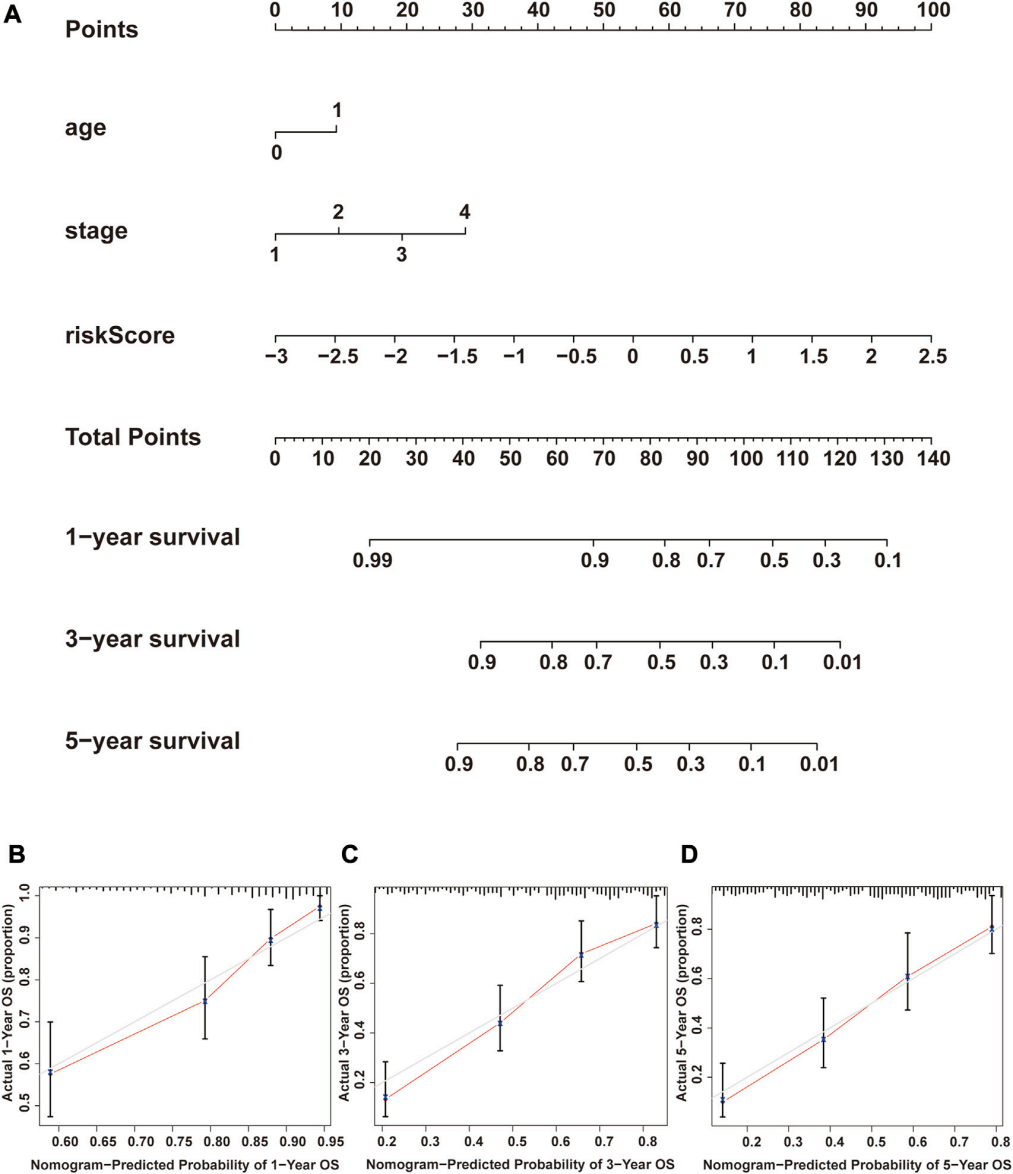
Construction and verification of the nomogram. **(A)** Nomogram combining clinicopathological variables and NRG score predicts 1-, 3-, and 5-year OS of BLCA patients. **(B–D)** Calibration curve test consistency between the actual OS rates and the predicted survival rates at 1, 3, and 5 years. N, lymph node; OS, overall survival.

We performed univariate Cox and multivariate Cox analyses to assess whether the NRG score was an independent prognostic indicator of BLCA patients. Univariate Cox regression analysis (Figure 8A) found that age, pathological stage, T-stage, N-stage, and NRG score were prognostic hazard factors. Multivariate Cox regression analysis (Figure 8B) pointed out that age (*p* = 0.024) and NRG score (*p* < 0.001) were independent prognostic indicators for BLCA patients. The ROC curves of the NRG score and clinicopathological variables were plotted, and the AUC results showed that the NRG score was a better independent prognostic factor (Figure 8C). Next, we established a novel prognostic nomogram on the basis of age, pathological stage, and NRG score (Figure 9A) to steadily and accurately predict the survival of BLCA patients in TCGA cohort. The calibration curves were used to assess the precision of the nomogram model (Figures 9B–D).

### Gene set enrichment analysis

**FIGURE 10:**
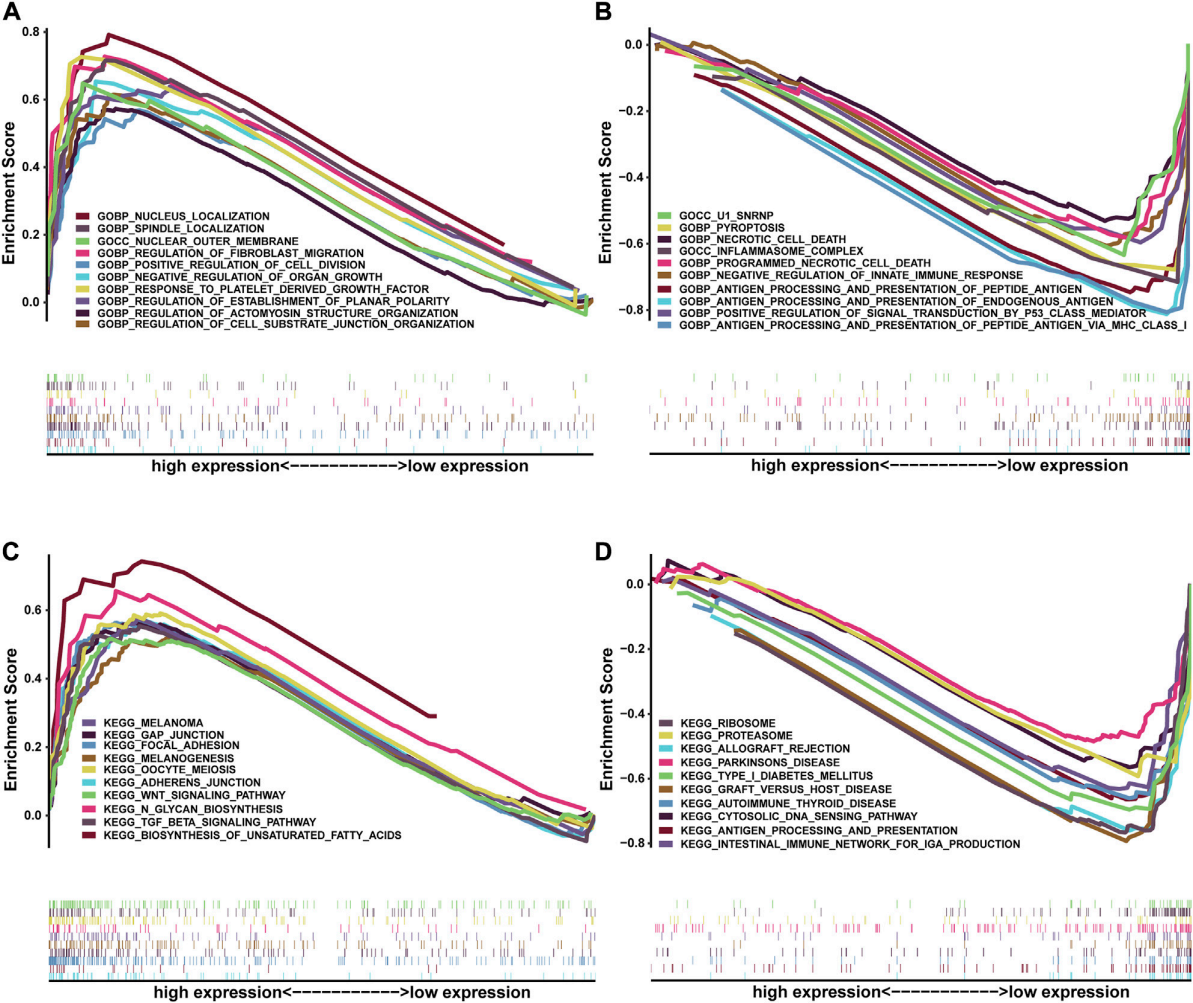
Correlation between NRG score and biological functions. GO-GSEA results of the high-risk group **(A)** and low-risk group **(B)**. KEGG-GSEA results of the high-risk group **(C)** and low-risk group **(D)**.

To further comprehend the effect of the NRG score on the biological characteristics of BLCA, we carried enrichment analysis of the prognostic signature in GO-GSEA (“pub/gsea/gene_sets/c5. go.v7.5.1. symbols.gmt”) (Figures 10A,B). In the high-risk group, we discovered that the pathways were mainly enriched in various biological regulations, such as organ growth, actomyosin structure organization, cell–substrate junction organization, establishment of planar polarity, and fibroblast migration. In addition, enrichment in the localization of the nucleus and spindle and the response to the platelet-derived growth factor of the high-risk group was conspicuous. As for cellular components, the high-risk group was enriched in the outer membrane, while the low-risk group is mainly concentrated in antigen processing and presentation of endogenous antigen, peptide antigen, and regulation of innate immune response and signal transduction. In addition, the cellular components of inflammasome complex and U1 snRNP were also enriched in the low-risk group. Furthermore, the enrichment of the KEGG-GSEA (“pub/gsea/gene_sets/c2. cp.kegg.v7.5.1. symbols.gmt”) showed that necroptosis-related genes were mainly enriched in the biosynthesis of unsaturated fatty acids and N-glycan in the high-risk group (Figures 10C,D). Meanwhile, the enrichment of signaling pathways of TGF-β, WNT and GAP junction and adherens junction was also presented. As for the low-risk group, major enrichment was found in multiple diseases such as Parkinson’s disease, autoimmune thyroid disease, type-1 diabetes mellitus, and graft-versus-host disease. In addition, this group was related to cytosolic DNA sensing pathways and antigen processing and presentation.

### Correlation between tumor immune microenvironment and NRG score

**FIGURE 11:**
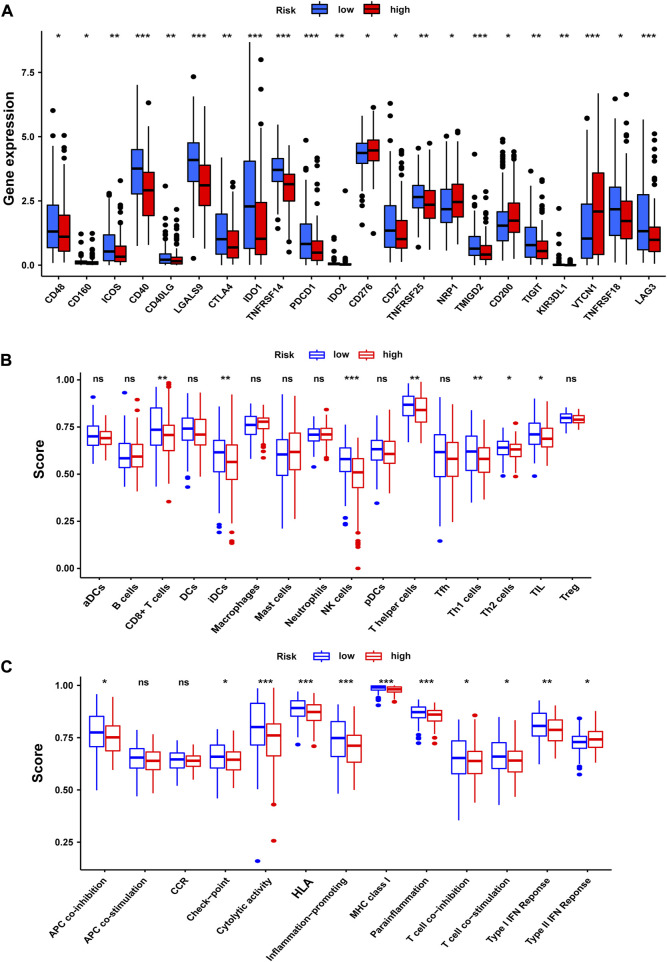
Tumor immune microenvironment of the NRG score. **(A)** Expression of immune checkpoints among high- and low-risk groups. **(B)** Infiltration of 22 TIICs in high- and low-risk groups. **(C)** Immune-related functions in the NRG score-defined groups. *p < 0.05; **p < 0.01; ***p < 0.001; ns = not significant.

For the importance of checkpoint-based immunotherapy, we analyzed the expression of immune checkpoints between the two groups as shown in Figure 11A. In our research, we discovered that the expression of CD48, CD160, ICOS, CD40, CD40LG, LGALS9, CTLA4, IDO1, TNFRSF14, PD-1, IDO2, CD27, TNFRSF25, TMIGD2, TIGIT, KIR3DL1, TNFRSF18, and LAG3 in the low-risk group was significantly higher than that in the high-risk group. Meanwhile, the expression of CD200, CD276, NRP1, and VTCN1 in the low-risk group was less than that in the high-risk group. In this study, we also performed a comparative analysis of immune cells to confirm the difference between high-risk and the low-risk groups (Figure 11B). The immune cell score of CD8^+^ T cell, immature dendritic cells (iDCS), NK cells, T helper cells, T helper type 1 (Th1) cells, T helper type 2 (Th2) cells, and tumor-infiltrating lymphocyte (TIL) in the low-risk group was higher than that in the high-risk group. We also evaluated the correlation of risk groups with immune-related functions. As shown in Figure 11C, the scores of APC (antigen-presenting cells) co-inhibition, checkpoint, cytolytic activity, HLA, inflammation-promoting, MHC-I, para-inflammation, T-cell co-inhibition, T-cell co-stimulation, and type-I IFN response in the low-risk group were significantly higher than those in the high-risk group. The score of type-Ⅱ-IFN response in the high-risk group was higher than that in the low-risk group. These findings pointed out that the NRG score was associated with the tumor immune microenvironment.

### Correlation between the NRG signature and drug Sensitivity

**FIGURE 12:**
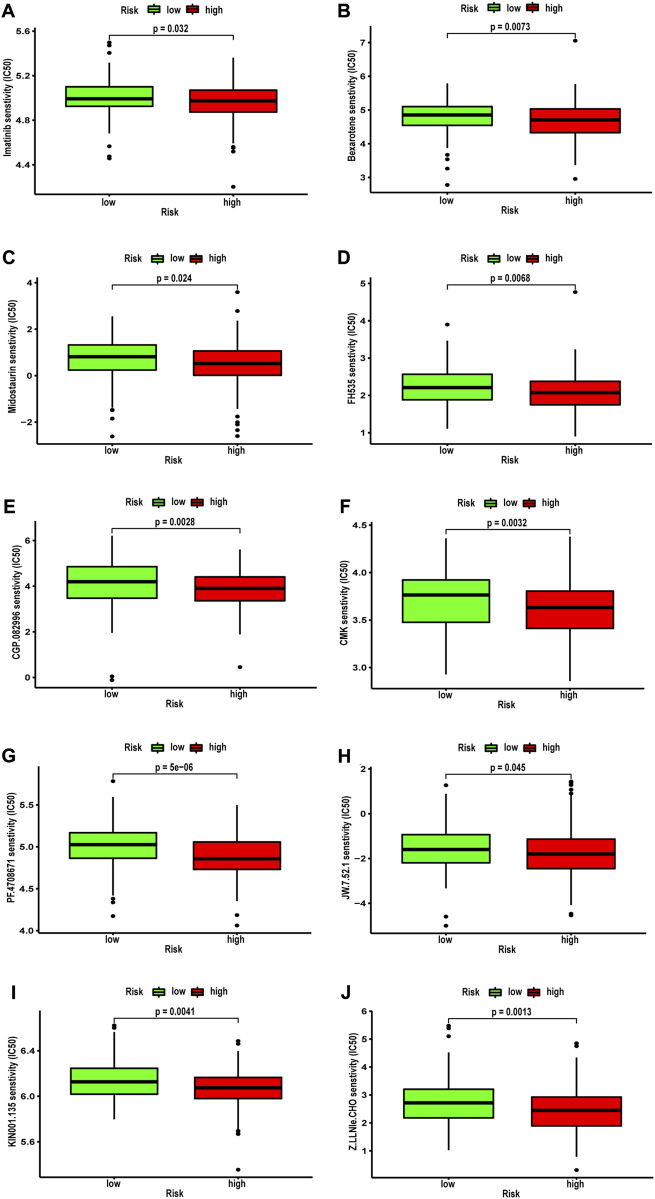
Correlation between NRG score and IC_50_ values of antitumor drugs, including **(A)** imatinib, **(B)** bexarotene, **(C)** midostaurin, **(D)** FH535, **(E)** CGP.082996, **(F)** CMK, **(G)** PF.4708671, **(H)** JW.7.52.1, **(I)** KIN001.135, and **(J)** Z.LLNle.CHO.

**FIGURE 13:**
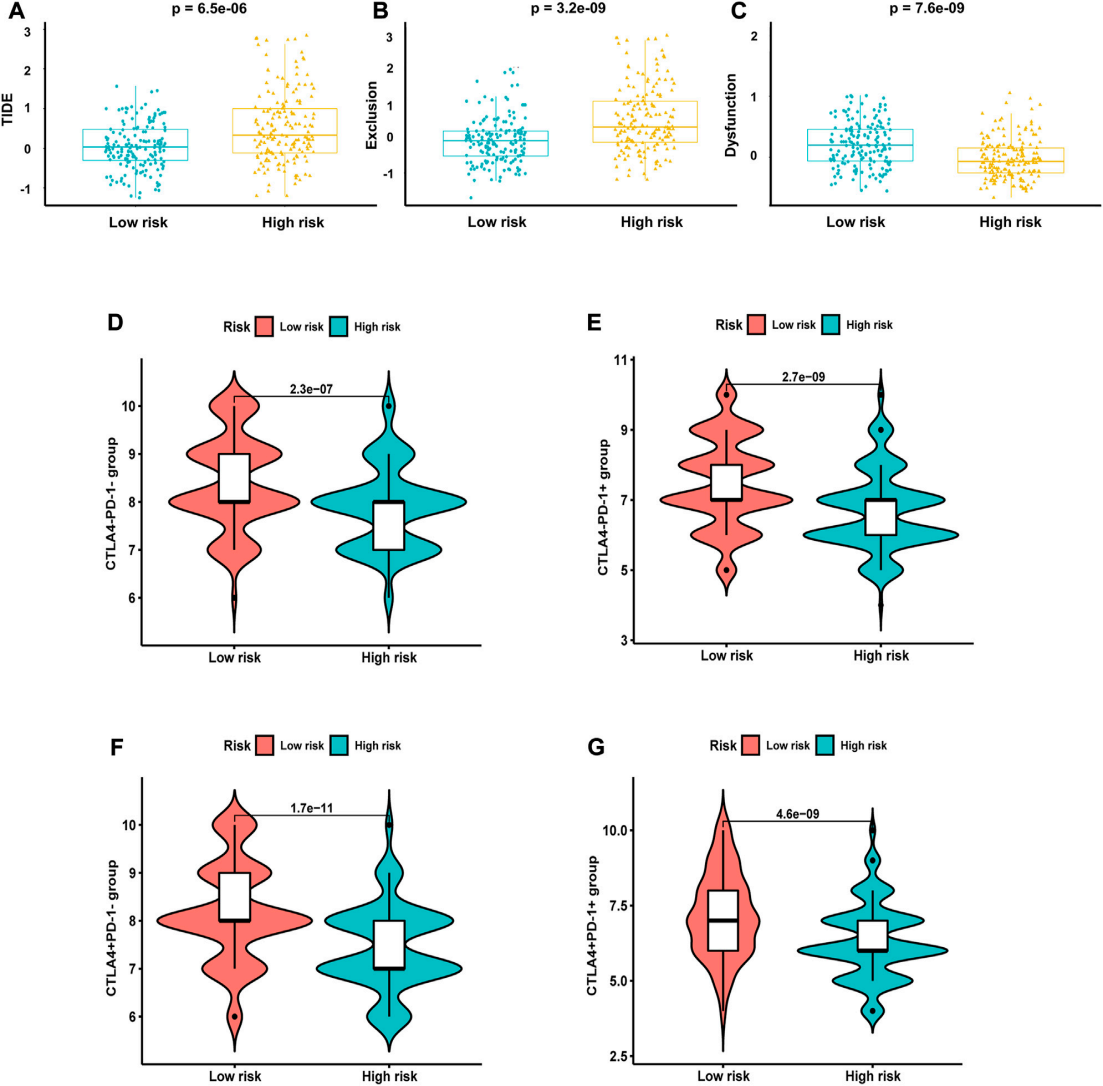
Response of different risk groups to immunotherapy. **(A)** TIDE score. **(B)** Exclusion score. **(C)** Dysfunction score. Immunophenoscore from TICA including **(D)** CTLA4-PD-1- group, **(E)** CTLA4-PD-1+ group, **(F)** CTLA4+PD-1- group, and **(G)** CTLA4+PD-1+ group.

Next, we analyzed the correlation between NRG score and the sensitivity of different antitumor drugs between the high-risk and the low-risk groups. We found that the high-risk group had lower IC_50_ values among various small-molecule targeted drugs (Figures 12A–J), such as imatinib, bexarotene, midostaurin, FH535, CGP.082996, CMK, PF.4708671, JW.7.52.1, KIN001.135, and Z. LLNle.CHO. These results suggested that patients in the high-risk group are more sensitive to these drugs, which may be used as a combination therapy drugs for the high-risk BLCA patients. We also compared the response to immunotherapy in different risk groups. As shown in Figures 13A–C, the TIDE score and exclusion score were lower in the low-risk group, and the dysfunction score was lower in the high-risk group. When we assessed the immunophenoscore of different risk groups from TICA website, including CTLA4-PD-1- group, CTLA4-PD-1+ group, CTLA4+PD-1- group, and CTLA4+PD-1+ group, we found that the low-risk group obtained higher immunophenoscore under all these conditions (Figures 13D–G, all *p* < 0.001). These results suggested that patients in the low-risk group may be more sensitive to immunotherapy.

## Discussion

Bladder cancer is a highly malignant urological tumor, and even BLCA patients with lower T stage (T1) have a recurrence rate as high as 23.5% in 5 years after the treatment of transurethral resection of the bladder tumor (Divrik et al., 2010). Although the diagnosis and treatment of BLCA have improved in recent years, the prognosis of BLCA patients is not satisfactory. Due to the high heterogeneity of BLCA, different BLCA patients often show different prognoses. Previous studies suggested that about half of BLCA patients do not benefit from first-line treatment regimens based on combination therapy with gemcitabine and cisplatin, and only 30% of BLCA patients achieve remission from ICI therapy. The tolerance of tumor cells to apoptosis leads to the resistance of cisplatin, leading to chemotherapy failure (Johnstone et al., 2002). Recent studies suggest that promoting immunogenic death may alter TME and TIL infiltration, and the combination of its inducers and ICIs acts synergistically in enhancing antitumor effects (Niu et al., 2022). Despite accumulating evidence that triggering necroptosis may serve as an alternative method for killing cancer cells, especially those that are not susceptible to apoptosis, research on necroptosis in bladder cancer remains less (Wang et al., 2022). Therefore, we conducted a comprehensive study of the role of necroptosis in BLCA in the present study.

First, we identified 17 NRGs associated with BLCA prognosis by analyzing 598 genes involved in necroptosis pathways using RNA sequencing results of 335 bladder patients from TCGA database and constructed two clusters by consensus clustering analysis. Necroptosis C2 showed a significantly better OS survival rate, and the proportion of BLCA patients in the early clinicopathological (<T2) stage and low grade of low-risk necroptosis C2 was also significantly higher. More importantly, the differential genes of C2 and C1 were mainly related to the immune response according to GO and KEGG analyses, especially in complement activation and TGF-beta signaling pathway. As we know, complement conducts immune surveillance and inhibits tumor progression through regulation of the immune system and direct killing of tumor cells (Klitgaard et al., 2013; Pokrass et al., 2013). The TGF-beta signaling pathway is a tumor suppressor that inhibits cell proliferation in the early stages of tumors by triggering cell arrest and apoptotic programs in cancer cells (David Charles et al., 2016).

Next, we constructed a novel NRG signature consisting of *SIRT6*, *FASN*, *GNLY*, *FNDC4*, *SRC*, *ANXA1*, *AIM2*, and *IKBKB* to predict the survival of TCGA-BLCA cohort (NRG score). Based on the NRG score, survival analysis showed that the low-risk group obtained a significantly better OS. In addition, the AUC analysis suggested that NRG has a good predictive value for BLCA patients’ prognosis. Univariate Cox regression and multivariate Cox regression analyses also confirmed that the NRG score could be considered as an independent prognostic risk, which was significantly proven in two separate external cohorts, namely, GSE13507 and GSE31684. Among the eight NRGs included in the NRG score, *ANXA1*, *FASN*, and *FNDC4* were upregulated and *GNLY*, *AIM2*, *SRC*, *IKBKB*, and *SIRT6* were downregulated in the high-risk group. Recent studies proved that targeting *ANXA1* abrogated Treg-mediated immune suppression in triple-negative breast cancer, and *ANXA1* was considered a worse prognostic and TME marker in gliomas (Bai et al., 2020; Lin et al., 2021). *FASN* is abnormally expressed in lots of tumors and is highly related to tumor migration and invasion (Zhang et al., 2021). Deng et al. (2021) found that downregulating the expression of FASN results in blockage of *de novo* fatty acid synthesis to inhibit the growth of bladder cancer. *FNDC4* promotes the tumor invasiveness of hepatocellular cancer as an extracellular factor (Wang et al., 2021). Also, among these downregulated genes, Jiang et al. (2020) suggested that a signature, including *GNLY*, was an ideal biomarker for predicting the better prognosis of MIBC patients. Fairfax et al. (2020) proved that *GNLY* was also associated with the cytotoxicity and characteristic of effector memory T cells in metastatic melanoma patients. Fukuda et al. (2021) confirmed that *AIM2* regulates anti-tumor immunity and is a viable therapeutic target for melanoma. Both *GNLY* and *AIM2* have been used to predict the prognosis of bladder cancer patients (Jiang et al., 2020; You et al., 2022). Low *SIRT6* expression is associated with poorer OS in gastrointestinal cancer (Wu X. et al., 2022). Wu et al. (2014) reported a loss of expression of *SIRT6* when muscle-invasive urothelial carcinoma of the bladder progresses from T2 to T4 stage, indicating more reliance on glycolysis when bladder cancer invades deeper through the bladder and into the adjacent tissues. However, there are few studies on the relationship between SRC, IKBKB, and tumors. In addition, in order to more accurately and conveniently predict the BLCA patients’ OS, we constructed a nomogram combining NRG score and several clinicopathological features.

Considering that necroptosis is different from apoptosis, which can release cellular contents to trigger a strong immune response, we investigated the correlation between NRG score and TME. GSEA shows that the low-risk group is mainly concentrated in antigen processing and presentation of endogenous antigen, peptide antigen, and the regulation of innate immune response and signal transduction, which will contribute to the anti-tumor effect (Balasubramanian et al., 2022). In the low-risk group, the infiltration levels of CD8 T cells, NK cells, and iDC cells were significantly increased, which always play important anti-tumor protective roles (Ma et al., 2013; Shimasaki et al., 2020; Raskov et al., 2021). Furthermore, classical immune checkpoints, such as CTLA4, PD-1, TIGIT, and LAG3, were also enriched in the low-risk group, and the analysis of TIDE also proved that the low-risk group obtained higher immunophenoscore under all these conditions, which reminds us that the application of ICIs in the low-risk group may achieve better therapeutic effects.

We also assessed the value of the NRG score to predict the sensitivity of chemotherapy and targeted agents in BLCA patients. The results showed that imatinib, bexarotene, and midostaurin benefited more in high-risk patients, and these drugs are generally used in the treatment of blood cancers. Furthermore, small molecules, including imatinib, bexarotene, and midostaurin, FH535, CGP.082996, CMK, PF.4708671, JW.7.52.1, KIN001.135, and Z. LLNle.CHO also had more significant benefits in high-risk patients. Recent research confirmed that targeting necroptosis with small molecules is a promising strategy for cancer therapy, which may be the key for overcoming apoptosis resistance, and has advantages in overcoming apoptosis resistance and stimulating antitumor immunity (Wu J. et al., 2022). Therefore, we believe that the NRG score can help identify better treatment strategies for individual patients with advanced BLCA.

The present study still has some limitations. First, this research is a retrospective study, and the clinical information of BLCA patients inevitably produces biases. Large, follow-up, multicenter, prospective studies are needed to further confirm our results. Second, the accuracy of the NRG score model in predicting drug efficacy also needs to be confirmed by clinical trials. Finally, the specific molecular mechanisms of NRG in BLCA that we included in our study remain to be further explored, especially SRC and IKBKB.

## Conclusion

Based on comprehensive analyses, we conducted an NRG score with excellent performance in assessing the prognosis, clinicopathologic features, TME, and therapeutic sensitivity of BLCA patients, which could be utilized as a guide for chemotherapy, ICI therapy, and combination therapy strategy.

## Data Availability

The original datasets for this study can be found in the manuscript/Supplementary Material; further information can be obtained by contacting the corresponding authors.

## Abbreviations

BLCA: bladder cancer; GEO: Gene Expression Omnibus; GO: Gene Ontology; KEGG: Kyoto Encyclopedia of Genes and Genomes; TCGA: The Cancer Genome Atlas OS: overall survival; BP: biological process; CC: cellular component; MF: molecular function; NRG: necroptosis-related gene; ICI: immune checkpoint inhibitor; TME: tumor microenvironment; FPKM: fragments per kilobase of transcript per million; LASSO least absolute shrinkage and selection operator; AUC: area under the ROC curve; DEG: differentially expressed gene; Th1: T helper type 1; Th2: T helper type 2; iDCS: immature dendritic cells; TIL: immature dendritic cells.
